# Effects of synthetic silymarin-PLGA nanoparticles on M2 polarization and inflammatory cytokines in LPS-treated murine peritoneal macrophages

**DOI:** 10.22038/IJBMS.2021.59312.13161

**Published:** 2021-10

**Authors:** Mojgan Azadpour, Mohammad Morad Farajollahi, Hassan Dariushnejad, Ali Mohammad Varzi, Amir Varezardi, Mitra Barati

**Affiliations:** 1 Research Center of Pediatric Infectious Diseases, Institute of Immunology and Infectious Diseases, Iran University of Medical Sciences, Tehran, Iran; 2 Department of Medical Biotechnology, School of Allied Medical Sciences, Iran University of Medical Sciences,Tehran, Iran; 3 Department of Medical Biotechnology, Faculty of Medicine, Lorestan University of Medical Sciences, Khorramabad, Iran; 4 Razi Herbal Medicines Research Center, Lorestan University of Medical Sciences, Khorramabad, Iran

**Keywords:** Cytokines, Nanoparticles, Peritoneal macrophages, PLGA compound, Silymarin

## Abstract

**Objective(s):**

Silymarin (SM) is a natural antioxidant compound with good anti-inflammatory effects, but its poor water solubility restricts its usage. Today, nanomaterial compounds (such as PLGA Poly D, L-lactic-co-glycolic acid) can provide a proper drug delivery system and help improve the accessibility of bioactive compounds to cells and tissues.

**Materials and Methods:**

In this study, PLGA nanoparticles (NPs) containing SM (SM-PLGA) were synthesized and characterized and their biological effects were evaluated on M2 macrophage polarization to regulate inflammation. SM-PLGA NPs were fabricated by the oil in water emulsion (O/W) method. Macrophages (MQs) were isolated from mouse peritoneum by the cold RPMI lavage protocol. Primary mouse MQ cells were treated by SM and SM-PLGA NPs and then stimulated with lipopolysaccharide (LPS). M2 polarization was evaluated by measurements of cytokine secretion levels (TNF-α, IL1-β, and IL-10), flow cytometry markers (F4/80, CD11b, CD38, and CD206), and the expression of specific proteins (M2 Ym1 and Fizz1).

**Results:**

SM-PLGA characterization showed that NPs were fabricated in the desired form. SM and SM-PLGA decreased pro-inflammatory cytokines (TNF-α and IL1-β) and increased IL10 as an anti-inflammatory cytokine. On the other hand, the M2-associated markers and proteins increased following treatment with SM and SM-PLGA. Post-hoc analysis indicated that these changes were more pronounced in the SM-PLGA group.

**Conclusion:**

This study revealed that SM-PLGA could markedly promote M2 polarization, thereby providing a valuable medical approach against sepsis and septic shock.

## Introduction

Sepsis is a clinical syndrome with a potentially life-threatening situation caused by the host immune response to an infection. In the sepsis process, the immune response plays a crucial role, and the storm of cytokines leads to an uncontrolled inflammatory response (1). It is estimated that 30 million people are annually affected by sepsis with high rates of morbidity and mortality (2). Despite progress in modern medicine, the treatment for sepsis is still a problem in health systems. Therefore, there is an urgent need to develop new drugs and strategies to cure this syndrome (3).

Macrophages (MQ) have a significant role in the pathogenesis of sepsis. As a routine process, microbial products such as lipopolysaccharide (LPS) trigger macrophage (M1) activation. The high secretion levels of pro-inflammatory cytokines, such as tumor necrosis factor-alpha (TNF-α), interleukin-6 (IL-6), and interleukin-1 beta (IL-1β), result in inflammation and organ failure (4). On the other hand, MQ could be converted to M2, which in turn leads to up-regulation of the anti-inflammatory response and tolerant protection against organ and tissue injury caused by M1 MQ (5). Based on the mentioned fact, induction of M2 polarization may provide a potential therapeutic approach to suppress the inflammation (6). 

Silymarin (SM) is one of the major ten natural products extracted from milk thistle (*Silybum marianum* L. Gaertn) seeds (7). SM is a complex of several components, mainly isosilybin A, isosilybin B, silybin A, silybin B, and other flavonolignans such as neosilyhermin, silychristin, silydianin, and silyhermin (8). Many investigations have indicated that SM possesses anti-inflammatory, anti-free-radical, glutathione depletion, anti-cancer, and anti-hepatitis properties (9). It has been reported that SM imposes its anti-inflammatory function by suppressing the major pro-inflammatory cytokines via NF-κB inhibition (10).

Today, nanotechnology provides fascinating vehicles for drug delivery (11). NPs have unique physicochemical properties that improve the bioavailability of therapeutic agents and enhance the efficacy of drugs (12). 

Polymeric NPs such as PLGA (Poly D, L-lactic-co-glycolic Acid), are used in drug delivery systems due to their biocompatibility and biodegradability (13). PLGA is an FDA-approved efficient nano-polymer that is used in the delivery of various drugs (13).

Despite the beneficial effects of flavonoids, their low water solubility is the main obstacle in using these agents in biomedical applications (14, 15). In this study, SM was loaded in PLGA and its characteristics determined afterward. Moreover, the SM-PLGA effects on M2 polarization were evaluated in LPS treated murine peritoneal macrophages, along with testing the possible involvements of SM in the secretion of pro- and anti-inflammatory cytokines.

## Materials and Methods

***Preparation of encapsulated SM in PLGA***

SM was loaded in PLGA Resomer RG 502 H, lactide: glycolide 50:50, Mw 7,000-17,000 NPs using emulsification and the solvent evaporation method (16). 

Briefly, 20 mg of SM (Sigma, USA) was dissolved in 2 ml of ethanol. This solution was mixed and stirred with 200 mg of dissolved PLGA in 5 ml of dichloromethane. The SM/PLGA mixture was added dropwise to 15 ml of 3% polyvinyl alcohol (PVA), MW 72000 solution and vortexed vigorously. It was then sonicated at 54% amplitude with a probe sonicator instrument (Hielscher, Germany) within an ice-bath for 2 min to form the oil in water emulsion, and the sonication was repeated 3 times to reduce the nanoparticle size. The prepared emulsion was immediately added to 0.3% PVA w/v solution. After this step, the aqueous phase was removed by stirring for 24 hr, the precipitated NPs were obtained by centrifugation and washed two times by PBS (15000 RPM, 30 min at 4 °C). Finally, SM-PLGA NPs were dissolved in sterilized water, freeze-dried, and lyophilized for 24 hr on an ATR FD 3.0 system (ATR Inc., USA) (16).

***Characterization of synthesized SM- PLGA NPs***

*Particle size and zeta potential*

The samples were diluted with deionized water (0.01%, w/v). The size profile of the SM- PLGA NPs was obtained by dynamic light scattering (DLS) analysis, and the surface charge of synthesized NPs was measured using a zetasizer (HORIBA SZ-100, Japan) at 25 °C. 

*Scanning electron microscopy (SEM)*

An SEM (FE-SEM, TESCAN, Czech Republic) was used for the surface morphology of the SM-PLGA NPs. The lyophilized samples were spread over a conductive tape and fixed on a metallic stud.

*Energy-dispersive X-ray (EDX) analysis*

The elemental composition of the SM-PLGA NPs was analyzed by an energy dispersive X-ray spectrometer (EDS/EDX) equipped with an SEM apparatus.

*Fourier-transform infrared (FT-IR) spectroscopy*

Functional group characterization was performed via the FTIR spectroscopy analysis using Unicom BRUKER (Taiwan). The FTIR spectrum was achieved from the sample spreading on the potassium bromide tablet.

***Determination of drug loading efficiency (DL)***

The drug loading of the PLGA NPs was calculated by analyzing precipitated nanoparticles. The SM-PLGA NPs were dissolved in 70% (v/v) methanol to disrupt the PLGA nano-polymeric structure. After that, the released SM from the NPs was determined using a UV–vis spectrophotometer (Shimadzu, Japan) at 288 nm, i.e., the wavelength of maximum absorption of SM in methanol (16). 

The percentage of drug loading (DL) in PLGA NPs was calculated as follows:

$\mathrm{DL\%}=\frac{\mathrm{weight of drug in Nanoparticles}}{\mathrm{weight of Feeding Drug}}\times 100$

***In-vitro drug release***

To determine the release kinetics of SM from SM-PLGA NPs, a certain amount of SM-PLGANPs containing 1 mg of SM was placed in a dialysis bag with a molecular weight of 12 kDa. The dialysis bag was immersed into 50 ml of PBS (pH 7.4) at 37 ± 0.5 °C with a stirring speed of 150 rpm. At certain intervals (0, 1, 2, 3, 4, 6, 8, 12, and 24 hr), 500 µl of the incubation medium was taken and replaced with fresh PBS. The amount of released drug in the PBS was measured by a UV–vis spectrophotometer (Shimadzu, Japan) at 326 nm, i.e., the wavelength of maximum absorption of SM in PBS (17).

***Isolation of murine peritoneal macrophages***

Primary murine peritoneal macrophages were isolated from male, 6–8 week-old, 18–22 g Balb/c mice, which were anesthetized and exterminated by rapid cervical dislocation. Euthanized mice were peritoneally injected with 5 ml of ice-cold RPMI medium. The mice abdomens were massaged gently to ensure the suspension of cells in the lavage fluid. An 18-G needle was used to slowly aspirate the lavage fluid from the peritoneal cavity. The peritoneal fluid was transferred to a 15-ml Falcone tube and kept on ice until centrifugation at 300 × g at 4 °C for 8 min. The supernatant was discarded, and the cell pellet was suspended in complete RPMI1640 with 10% fetal bovine serum (FBS). The suspension was transferred to T25 tissue culture flasks and incubated in 5% CO_2_ at 37 °C for 4 hr. Non-adherent cells were removed by gently washing 3 times with warm PBS (18). The cells were cultured as primary macrophages and used for the next experiments.

***Cell cytotoxicity assay***

The cytotoxicity effect of the SM and SM-PLGA NPs on MQ isolated from murine peritoneum was examined by the MTT assay. Briefly, 1×10^5^ of the MQ cells were seeded in 96-well plates and incubated overnight. The culture medium was replaced with 200 µl of SM diluted in DMSO 10%, and SM-PLGA NPs were diluted in complete RPMI1640 medium at final concentrations of 3.9, 7.8, 15.6, 31.2, 62.5, 125, and 250 μg ml^−1^. After 24 hr, 10 μl of MTT solution (5 mg/ml) was added to each well and incubated at 37 °C for 4 hr. Subsequently, the supernatant media were replaced with 100 μl of DMSO to dissolve the formazan crystals. The optical density was recorded at 570 nm using a microplate reader (STAT-FAX, USA). 

Cell viability assessment was calculated by the following equation:

$\mathrm{Cytotoxicity\%}=1-\frac{\mathrm{Absorbance Test}}{\mathrm{Absorbance Control}}\times 100$

The IC_50_ values (concentrations that induced 50% cytotoxicity) were calculated by a regression using SPSS software version 19 (SPSS Inc., USA).

***Pro-inflammatory activation of primary macrophages***

**Figure 1:**
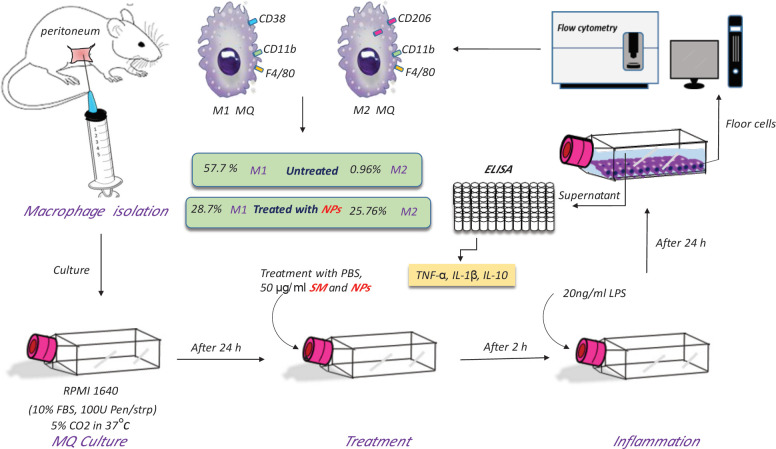
Macrophage isolation, stimulation, and M2 polarization measurement process

The pro-inflammatory activation of primary macrophage cells was performed based on a method described by Rusmana *et al*. (19). The cells were seeded in a 6-well plate at a density of 10^5^ cells per well and incubated at 37 °C overnight (in 5% CO_2_ and humidified atmosphere). Then, the medium in the wells was replaced with fresh medium 2 hr before the LPS treatment. SM and SM-PLGA NPs were added to separate wells at a final concentration of 50 µg/ml. Next, 200 µl of LPS (Sigma Aldrich, USA) at 100 ng/ml concentration was added to each well, and the plates were incubated for 24 hr. Untreated wells were considered negative control, and those that only received LPS were considered positive control. After the incubation period, centrifugation was performed at 2000 rpm for 10 min, and the supernatant was collected for cytokine measurement assays. The cells were detached and kept for further experiments (Figure 1).

***M2 polarization measurement***

*Flow cytometry analysis*

The treated macrophages were harvested and washed with FACS buffer (PBS with 2% FBS and 1 mM of EDTA) twice. Macrophage cells were blocked with anti-mouse FcR antibody (CD16/32, clone 93, Biolegend) in FACS buffer at 4 °C for 15 min. Then, the cells were stained with antibodies for CD11b (clone M1/70, PE/cy5, Biolegend), F4/80 (clone BM8, FITC, Biolegend), CD206-APC, and CD38 (clone 90, APC, Biolegend), or isotype control at 4 °C for 15 min and analyzed on a BD FACSCalibur2 Laser Flow-Cytometer (BD, USA). Flow cytometric data were analyzed using the FlowJo software (Tree Star, OR).

*Cytokine measurement by Enzyme-linked immunosorbent assay (ELISA)*

The collected supernatant of treated macrophages was used to measure TNF-α, IL-1β, and IL-10 by the ELISA assay (Invitrogen, USA) according to the manufacturer’s instructions.

*Western blot*

Macrophages were treated with SM, SM-PLGA NPs, and positive and negative controls for 24 hr. Cells were lysed in an ice-cold Radioimmunoprecipitation assay (RIPA) buffer containing protease phosphatase inhibitors. The extracted proteins were quantified by a bicinchoninic acid protein assay kit (Pars, Iran). The proteins (80 μg) from the whole cell lysate were separated on 10% SDS-PAGE gel and transferred to a methanol-preactivated polyvinylidene fluoride (PVDF) membrane (BIORAD, USA). The membranes were probed with primary antibodies against FIZZ1 and Ym1 proteins (R&D Systems, Inc, USA) and incubated at 4 °C overnight. After washing, the membrane was incubated with a secondary antibody conjugated with horseradish peroxidase (Sigma, Germany). The immune complex was visualized using an enhanced chemiluminescence kit (Clarity western ECL kit; Bio-Rad, USA). The protein level was analyzed using Image J software (National Institute of Health, Bethesda, MD, USA).

***Statistical analysis***

All data were obtained in three independent experiments and analyzed statistically using SPSS software version 19 (SPSS Inc., USA). Statistical differences were assessed by one-way analysis of variance (ANOVA), and a value of *P*<0.05 was considered significant. All graphs were depicted with the Graph Pad Prism 6.01 software.

## Results

***Preparation and characterization of SM-PLGA NPs***

SM is a poorly water-soluble agent that was encapsulated in PLGA NPs for enhancing bioavailability. The SM-containing PLGA NPs were fabricated using PVA according to conventional emulsification/solvent evaporation procedures. In synthesizing the PLGA nano-polymer, dichloromethane and PVA functioned as the solvent and stabilizing agents, respectively. The formulation was optimized at 3% PVA concentration, 3 min sonication time, and a 1:10 ratio of SM to PLGA. This optimization resulted in appropriate particle size with optimum entrapment. 

*DLS, zeta potential, and drug loading efficiency (DL)*

*analyses*

DLS determined the size distribution of the SM-PLGA NPs. The average particle diameter was 180 ± 20.6 nm at 25 °C with narrow size distribution and a polydispersity index (PDI) of 0.226 ± 0.036.

The measured zeta potential of PLGA nano-polymer and SM-PLGA samples indicated surface charges of −12.8 and −17.6 mV for PLGA-NPs (blank) and SM-PLGA, respectively.

The DL of SM in PLGA was calculated at 86.43±6.43% using the equation mentioned in the Materials and Methods section.

*Scanning electron microscopy (SEM) analysis*

**Figure 2:**
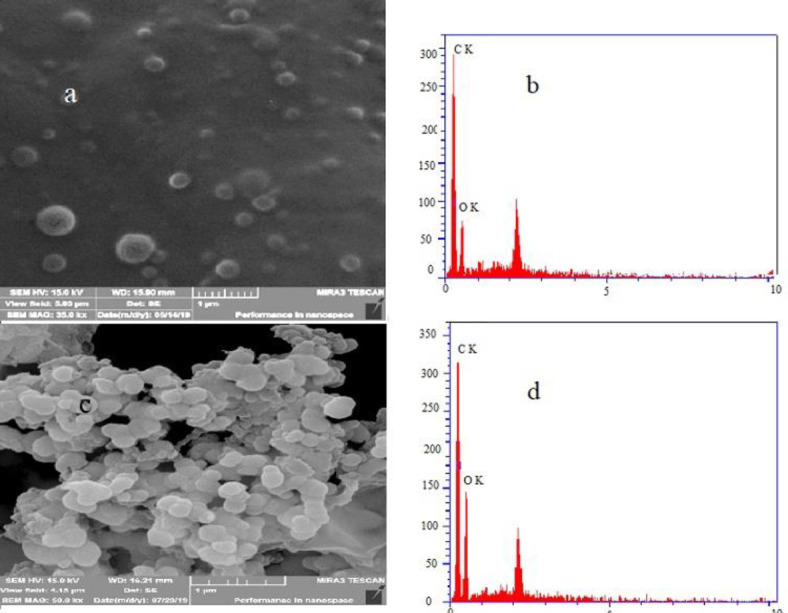
Scanning electron microscope photograph and EDX (X-ray diffraction) of blank (a, b) and silymarin- PLGA NPs (c, d)

The NPs were observed by an SEM to evaluate their physicochemical characterization. The results of microscopic images of blank and SM-PLGA are shown in Figure 2.

As illustrated in Figure 2, the PLGA NPs are spherical and uniform, with a size ranging from 115 to 154 nm (Figure 2a). After the encapsulation and formation of SM-loaded PLGA, the particle sizes ranged between 152 and 193 nm (Figure 2c), suggesting the significantly improved dispersion of NPs.

According to Figure 2b (blank sample) and Figure 2d (SM-PLGA NPs), the analysis of X-ray diffraction (EDX) spectrometry showed that the carbon mass in NPs (68.30 %) was more than the blank sample (59.30), indicating the SM loading in PLGA NPs. 

*FT-IR analysis*

**Figure 3:**
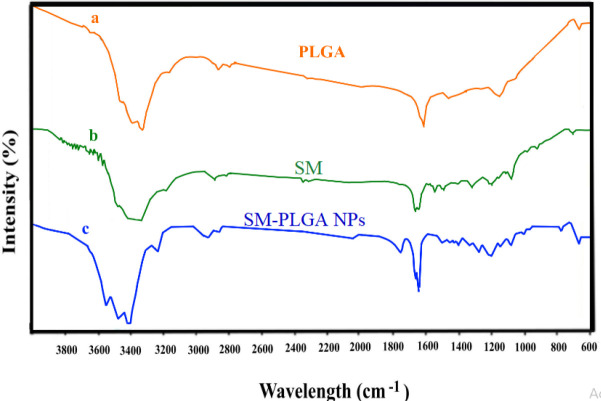
FT-IR spectrum (a) PLGA, (b) Silymarin (SM), and (c) SM-PLAG NPs

The functional groups were detected by FT-IR analysis. NPs were scanned in the range of 4000–600 cm^−1^, and recording the spectra showed various peaks in NPs. The FT-IR spectrums of PLGA and SM-PLGA are shown in Figure 3. In the FTRI record, PLGA NPs show the sharp peak of a carboxyl group (C=O stretching vibrations) at 1730 cm^−1^ and a strong broad O-H stretching vibration around 3479 and 3553 cm^−1^. Additionally, the sharp absorption bands at 1638 (C O stretching) and the PLGA peak at 1618 cm^−1^ confirm the presence of SM in the SM- PLGA NPs. Moreover, the presence of a peak in SNPs at 1638 cm^−1^ corresponds to the C-C ring, stretching in SM, substantiating drug presence in the SM-PLGA NPs.

***In vitro drug release study***

**Figure 4:**
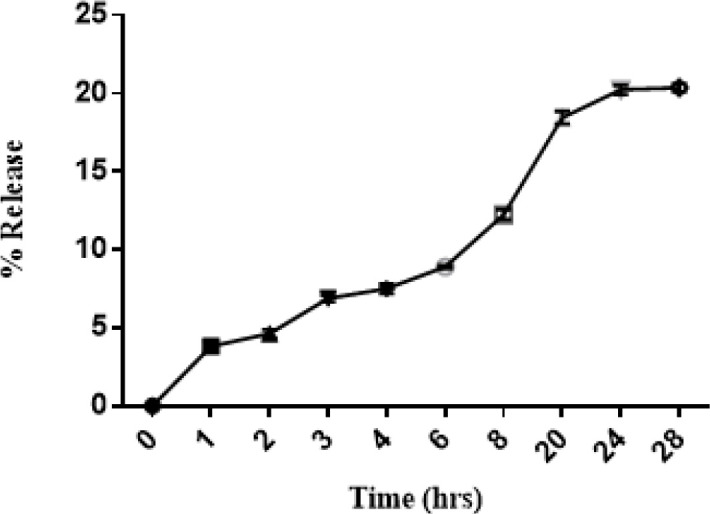
Release kinetics of Silymarin (SM) from PLGA nanostructure

The release study of SM from SM-PLGA NPs is shown in Figure 4. The SM-PLGA NPs exhibited an initial drug release of about 18% after 20 hr, and 20% of SM was released at 28 hr, after which the drug release became slower. 

***Cell viability and MTT-based cytotoxicity assay***

**Figure 5:**
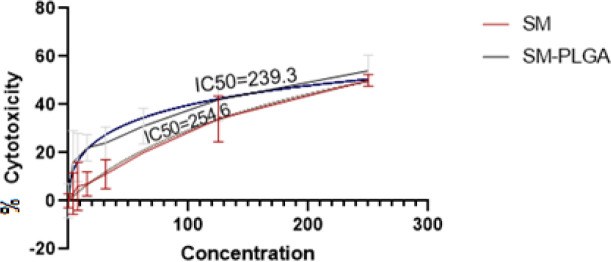
Macrophage cell cytotoxicity (%) treated with different concentrations of Silymarin (SM) and SM-PLGA NPs (ϻg/ml)

The cytotoxic effect following SM and SM-PLGA NPs treatment was evaluated on the isolated MQ by the MTT assay (Figure 5). The IC_50_ (inhibitory concentration 50) value was calculated for each case. At a low dose of 62.5 µg/ml, the treated cells showed no significant cytotoxic effect to SM and SM- PLGA. The IC_50_ values of free SM and SM- PLGA were respectively 254.6 and 239.3 μg/ml for 24 hr. Based on these results, a dosage of 50 μg/ml was chosen as the maximum dose concentration for both substances in the following experiments.

***M2 polarization measurement***

*Flow cytometry analysis*

MQs were categorized using flow cytometry. Membranes CD38 and CD206 were detected after gating with F4/80 and CD11b.

**Figure 6:**
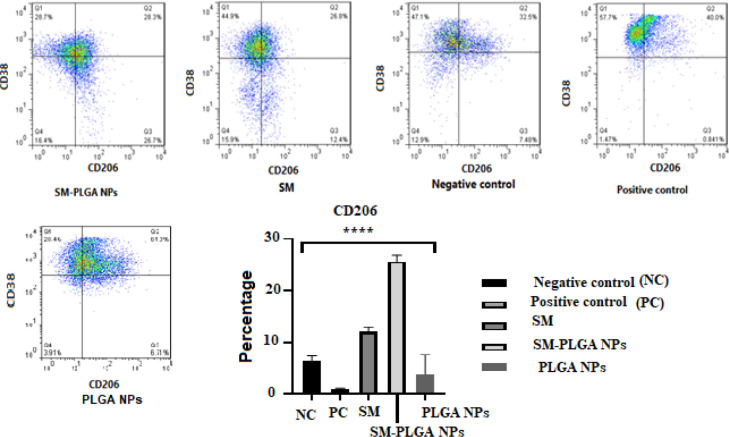
Flow cytometry analysis of MQ treated with PBS (negative control), LPS (Positive control), LPS and SM (Silymarin (SM) group), LPS and Silymarin-PLGA NPs (SM-PLGA NPs group), and PLGA NPs. In Positive control group CD206+ cells (M2) was 0.96 ± 0.2%, in Silymarin and Silymarin- PLGA NPs was 12.1 and 25.76 %, respectively. (**** *P*-value<0.01)

The cells that displayed CD38+ CD206- phenotype were considered M1, and those that demonstrated CD38- CD206+ phenotype were considered M2 MQ. In the positive control group (Figure 6). CD38 was expressed in more than 92.2 % ± 2.68 of cells, whereas less than 1% of these cells were positive for CD206 staining. In contrast, 18.4% ± 6.46 of negative control cells displayed the CD38+ CD206- phenotype, and less than 3% of cells displayed the CD206+ phenotype.

In SM and SM-PLGA NPs groups, the percentage of M2 phenotype cells (F4/80+CD11b+Cd38−CD206+) was much higher than negative and positive control groups (Figure 6).

*Cytokine assay*

**Figure 7:**
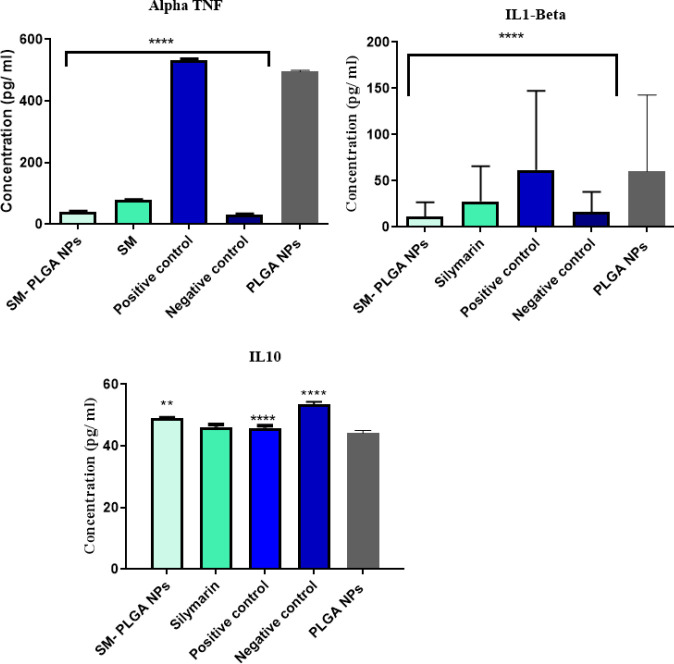
Cytokine measurement (TNF- α, IL1-β, and IL10) in MQ treated with PBS (negative control), LPS (positive control), LPS and SM (Silymarin Group), LPS and PLGA NPs (PLGA NPs), LPS and Silymarin-PLGA NPs (Silymarin- PLGA NPs). ** *P*-value<0.05, ****P-value<0.01

Pro- and anti-inflammatory cytokines were assessed after 24 hr of MQ stimulation with LPS (Figure 7). LPS induced the expression of TNF-α and increased its level. As shown in Figure 7, the TNF-α level was significantly higher in the positive control group (cells with LPS stimulation) than the negative control group (cells without LPS stimulation). SM and SM-PLGA NPs treatments had a significant inhibitory effect on the TNF-α production. The results of IL-1β in treatment groups showed a similar pattern. A subsequent *post-hoc* analysis (Table 1) revealed that TNF-α and IL-1β levels were significantly lower in the SM-PLGA NPs group than in the SM group (*P*-value<0.0001). After the stimulation with LPS, a decrease was observed in the IL-10 level, whereas it significantly increased in both SM-PLGA and SM groups. The level of IL-10 was significantly higher in SM-PLGA and SM groups than in other groups. Results of the *post-hoc* analysis indicated that the level of IL-10 was higher in the SM-PLGA NPs group than in the SM group (*P*-*value* = 0.001).

*Western blot*

**Figure 8:**
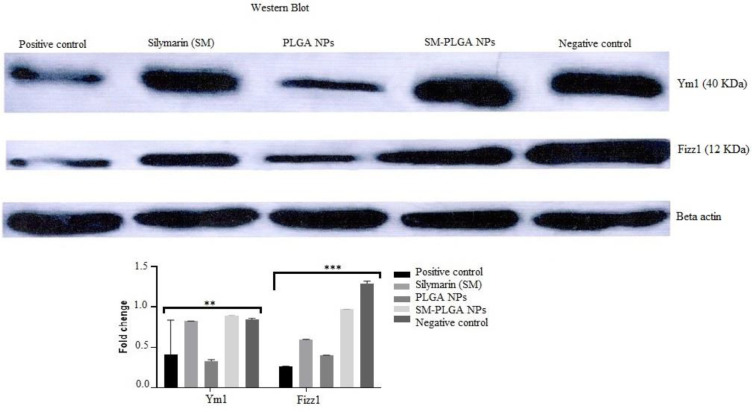
Western blot analysis of FIZZ1/YM1 expression in 50 ng of protein of cell lysate of stimulated cells by LPS alone (positive control), stimulated and treated by SM (Silymarin group), PLGA and SM-PLGA NPs, and normal cells without any stimulation and treatment (Negative control)

The expression levels of anti-inflammatory FIZZ1 and YM1 protein in MQ were determined by western blotting. The protein level increased in the sepsis-induced treatment group of SM and SM-PLGA NPs as compared with the positive control group for 24 hr after the induction by LPS. The enhancement amplitude was uppermost in the sepsis SM-PLGA NPs group (Figure 8).

**Table 1:** Post hoc analysis of (TNF- α, IL1-β and IL10) in MQ treated with PBS (negative control), LPS (positive control), LPS and SM (Silymarin Group), LPS and PLGA NPs (PLGA NPs), LPS and Silymarin- PLGA NPs (SM- PLGA NPs)

PLGA NPs	SM group	SM-PLGA	Positive group	Negative group	**Cytokine**
**495.48± 4.2** ^a^	**78± 0. 8** ^b^	**40.33± 0. 85** ^c^	**531.9± 3.6** ^a^	**31.7 ± 0. 64 ** ^d^	**TNF- α**
**118.65± 1.6** ^a^	**54.6± 0. 69** ^b^	**22.43± 0.54** ^d^	**122.32± 1.22** ^a^	**31.64± 0. 51** ^c^	**IL1**-ᵦ
**44.1± 0.91** ^c^	**46.14± 0. 28** ^c^	**49.01±0.10** ^b^	**45.85± 0.79** ^c^	**53.56± 0.76** ^a^	**IL10**

## Discussion

Findings of the *in vitro* tests with mouse peritoneal macrophages indicated that treatment with SM-PLGA NPs could increase the polarization of M1 mouse peritoneal macrophages to M2. Our data showed that treatment with SM and SM-PLGA NPs reduced the secretion of inflammatory factors (IL1-β and TNF-α) and increased the induction of FIZZ1, YM1 protein, and IL10 cytokine in LPs-stimulated macrophages. According to flow cytometry analysis, the expression of CD206 was significantly higher in the SM-PLGA NPs Group. In all assays, the findings of PLGA NPs were nearly identical to the positive control.

The innate immune system responses to bacteria and their products motivate the pathophysiology of inflammatory diseases such as sepsis (20). According to Lee’s research, sepsis produces anomalies in drug microsomal metabolism, particularly in the late stages, and is linked to high levels of oxidative stress and lipid peroxidation (21). Moreover, there is evidence that oxidative stress contributes significantly to the development of sepsis-induced multi-organ failures (22). The M2 subset of macrophages expressed anti-inflammatory agents, such as TGF-β and IL-10, to secure the balance between pro-and anti-inflammatory responses (23). Previous studies have shown that three profile markers for M2 polarization evaluation include F4/80, CD38, and CD206, the pro-and anti-inflammatory cytokines, and the expression of Ym1 and Fizz1 proteins (24-30). The IL-10 cytokine, FIZZ1, and Ym1 proteins are expressed by M2 MQ and affect the inflammatory response (31). The predominant macrophage subset regulation might be considered an effective treatment for sepsis and inflammatory disease (32). 

The therapeutic and anti-inflammatory properties of SM have been tested in various studies (33-36). SM has the potential to protect the cell membranes of the liver by inhibiting the production of NO and it is also an anti-lipid peroxidation and reactive oxygen species (ROS) removal agent from the body (37–39). Research revealed that SM could improve oxidative stress damage caused by benzo (a) pyrene and exogenous ROS (H2O2), as well as lipid peroxidation scavenging ability and antioxidant defense ability (38). The therapeutic effects of SM on rats suffering from sepsis caused by CLP were demonstrated by Cihan *et al.* (34) who found that TNF-α and IL-1 levels significantly decreased after treatment with SM. They also indicated that superoxide dismutase activity significantly decreased in the SM group compared with control and sepsis groups (34). 

The results of our study on DLS measurements showed that the hydrodynamic diameter of SM-PLGA NPs was less than 200 nm. The SM-PLGA NPs had a zeta potential of -17.6 mV, indicating a net negative surface charge and boosting nanoparticle stability while lowering nanoparticle self-assembly. The carboxylic group of PLGA exposed on the surface of the NPs could be responsible for the negative charge of NPs. The shape and surface morphology of SM-PLGA NPs evaluated by SEM revealed that the shape of the NPs was spherical and uniformly distributed with a smooth surface. The FTIR analysis of SM-PLGA NPs exhibited the typical carboxyl group (C O stretching vibrations) peak at 1730 cm^-1^, alkane stretch (C H stretching vibrations) at 2924, and strong broad O H stretching vibrations around 3416 and 3479 cm^-1^. In a study using the DLS analysis, the size of the synthetic SM-PLGA NPs (SNPs) was less than 300 nm, and the FT-IR analysis conﬁrmed the encapsulation of SM by the SNPs (40). These results are confirmed by characterization of SM-LPG NPs in the current study, but the size of NPs was smaller. Chu *et al.* reported that the inhibition of polarization of M2 macrophages in mice treated with INCB3344 (dimethyl sulfoxide/carboxymethylcellulose) had higher infarction volumes associated with reduced M2 polarization (41).

The CD38/CD206 flow cytometry assay discriminates between M1/M2 macrophage phenotypes. It has been reported that M1 MQs can be characterized by expression of CD38 using flow cytometry (31), and since CD206 is not expressed on the M1 macrophage, it is a useful marker for M2 macrophage detection (42). Our results demonstrated that treatment with SM-PLGA NPs led to more activation of mouse peritoneal macrophage polarization from M1 to M2 phenotype than the other groups. In 2015, the flow cytometric analysis in a study revealed that the XBJ (a traditional Chinese herbal mixture) group had a significantly higher percentage of M2 phenotypic macrophages than the control group after LPS stimulation *in vitro* (36). We found comparable results for SM-PLGA in the current investigation. The modulation of the JAK1-STAT6 signal pathway could be a viable mechanism to explain the beneficial effects of both SM and XBJ on the M2 shift.

A study (2018) revealed that azithromycin, tofacitinib, hydroxychloroquine, and pioglitazone had an anti-inflammatory profile as they enhanced some M2 markers (43).

Our findings revealed that treatment with SM and SM-PLGA NPs influenced the reduction of TNF-α and IL1- β. According to another study, SM could reduce LPS-induced IL-1β production in isolated mouse peritoneal macrophages and RAW 264.7 cells (33), which is similar to our results. Juma’a *et al*. reported that SM had anti-inflammatory activity in a murine chronic inflammation model (44). The anti-inflammatory effects of SM can be attributed to inhibition of the transcription nuclear factor kappa B (NF-ƙB) activation, in which NF-ƙB plays a role in the production of IL-1, IL6, IFN-ᵞ, and TNF-α (45).

Furthermore, we found that reduction of pro-inflammatory cytokines was more pronounced in the SM-PLGA NPs group than in the SM group. According to the previous findings, the encapsulation of SM into PLGA could improve water solubility, bioavailability, and subsequently, its anti-inflammatory properties (16). Therefore, the enhanced anti-inflammatory effect of SM-PLGA NPs can be explained through this mechanism. It is well known that one of the most significant anti-inflammatory cytokines, IL-10, inhibits the secretion of IL-6 and TNF-α (46). The auto-phosphorylation of the receptor after being linked to IL10 activates the STAT3 transcription factor and inhibits the expression of pro-inflammatory cytokines through its binding mediators (46). It is a Th2 product and a powerful inhibitor of Th1 cells (47).

Kim *et al*. showed that IL-10 caused an increase in the expression levels of mRNA and the AIM (apoptosis inhibitor of macrophage) protein in mouse bone marrow-derived macrophages (BMDM), and inhibition of STAT3 reduced the IL-10-induced AIM expression (48). Results of our study demonstrated that the level of IL10 as an M2 cytokine increased in treatment groups compared with the positive group. Moreover, the results of the *post-hoc* analysis indicated that the level of IL10 was higher in SM-PLGA NPs. In addition, we found that the expression levels of FIZZ1 and YM1 as the other M2 markers were up-regulated following SM and SM-PLGA therapy. These findings show that SM-PLGA shifted the M1 subtype of MQ to M2. Similar to our findings, a study indicated that XBJ could shift M1 to M2 associated with the JAK1-STAT6 signal pathway (36). The molecular mechanism of the anti-inflammatory effect of SM and SM-PLGA NPs is likely to depend on the SM antioxidant properties and the transcriptional modulation relevant to the JAK-STAT pathway. The immune regulation during different inflammatory diseases and macrophage polarization are affected by the JAK-STAT pathway (34). Previous studies indicated that the JAK1-STAT6 signal pathway was linked to the expression of M2-related molecules such as Arg1, CD206, Fizz1, and Ym1 (41). Blocking the activity of IL-1β by IL-1 receptor reduced the mortality of patients with sepsis (21). Tong *et al*. reported M2 macrophage bias induced by silibinin in RAW264.7 and found that silibinin significantly inhibited TNF-α-induced production of IL-6 and IL-1β in rheumatoid arthritisfibroblast-like synoviocytes (RA-FLS) (49).

## Conclusion

As SM has low solubility in water, encapsulation in PLGA could improve its accessibility for tissue and cells and enhance its anti-inflammatory effects. Treatment with SM-PLGA NPs switched M1 macrophages to M2, which is a valuable medical approach against sepsis and septic shock. The next stage of our research will evaluate the effects of treatment with SM-PLGA NPs on cecal ligation and puncture (CLP) as the mouse model of sepsis.

## Authors’ Contributions

MB Study concept and design and supervision of critical revision of the manuscript; MA Original idea, technical and material support, acquisition of data, statistical analysis, drafting of the manuscript; MMF Material support; AMV Administrative tasks; HD Critical revision of the manuscript for important intellectual content; and AV Drafting of the manuscript.

## Ethics Approval

 Compliance with ethical standards.

## Consent to Participate

Not applicable*.*

## Conflicts of Interest

The authors declare no conflicts of interest.
